# Supplementary material for: Insulin Downregulated the Infection of Uropathogenic Escherichia coli (UPEC) in Bladder Cells in a High-Glucose Environment through JAK/STAT Signaling Pathway
Source: Microorganisms. 2021 Nov 24;9(12):2421. doi: 10.3390/microorganisms9122421 (PMC8704104; doi:10.3390/microorganisms9122421)
Supplement: Supplementary file 1 [file microorganisms-09-02421-s001.zip › microorganisms-1417429-supplementary for final.pdf]

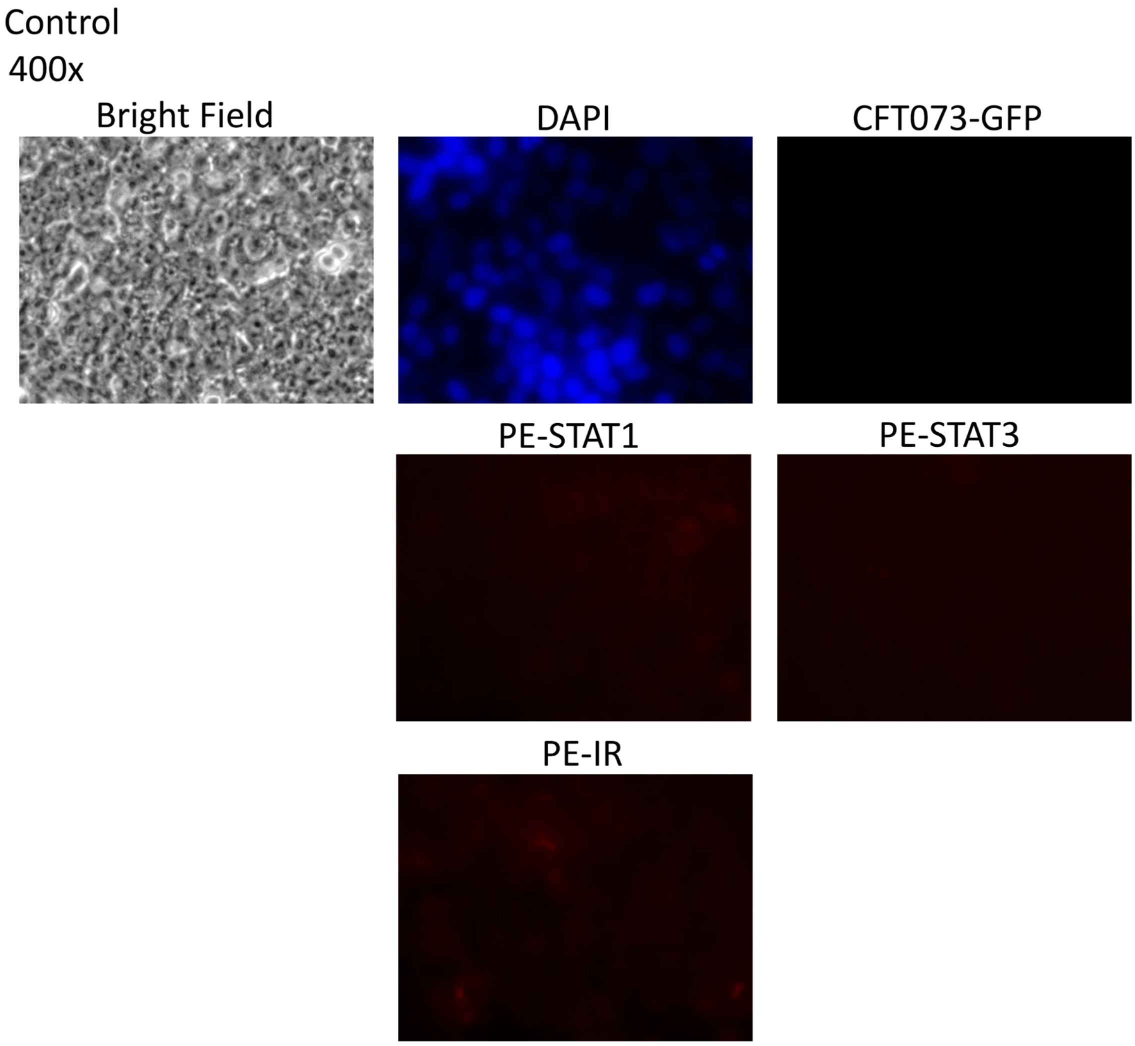

Figure S1. GFP-UPEC, PE-STAT1, PE-STAT3 and PE-IR expression images of SV-HUC-1 cells without treatment or UPEC infection as the negative control. DAPI was used as a standard for observing the fluorescence expression ratio of cells. Cell images were captured using a microscope (Leica) at 400 × magnification.
